# Modeling adsorption with lattice Boltzmann equation

**DOI:** 10.1038/srep27134

**Published:** 2016-06-03

**Authors:** Long Guo, Lizhi Xiao, Xiaowen Shan, Xiaoling Zhang

**Affiliations:** 1 State Key Laboratory of Petroleum Resources and Prospecting, China University of Petroleum, Beijing 102249, China; 2 Beijing Aeronautical Science and Technology Research Institute of COMAC, Beijing 102211, China; 3 Research Institute of Petroleum Exploration and Development, China National Petroleum Cooperation, Beijing 100083, China

## Abstract

The research of adsorption theory has recently gained renewed attention due to its critical relevance to a number of trending industrial applications, hydrogen storage and shale gas exploration for instance. The existing theoretical foundation, laid mostly in the early twentieth century, was largely based on simple heuristic molecular interaction models and static interaction potential which, although being insightful in illuminating the fundamental mechanisms, are insufficient for computations with realistic adsorbent structure and adsorbate hydrodynamics, both critical for real-life applications. Here we present and validate a novel lattice Boltzmann model incorporating both adsorbate-adsorbate and adsorbate-adsorbent interactions with hydrodynamics which, for the first time, allows adsorption to be computed with real-life details. Connection with the classic Ono-Kondo lattice theory is established and various adsorption isotherms, both within and beyond the IUPAC classification are observed as a pseudo-potential is varied. This new approach not only enables an important physical to be simulated for real-life applications, but also provides an enabling theoretical framework within which the fundamentals of adsorption can be studied.

Adsorption, and its counter process, *desorption*, are ubiquitous in nature and its understanding and modeling are crucial to many promising industrial applications ranging from the storage of gas fuel such as natural gas and hydrogen^1^ to the exploration of shale gases^2^. According to International Union of Pure and Applied Chemistry (IUPAC)^3^, the word *adsorption* describes “an increase in the concentration of a dissolved substance at the interface of a condensed and a liquid or gaseous phase due to the operation of surface forces”. In this process, a thin film of the dissolved substance, called *adsorbate*, forms on the surface of the condensed phase, called *adsorbent*, in contrary to the similar of *absorption* in which the adsorbate *permeates* into the bulk of the absorbent. It has been known for a long time that adsorption happens due to the interaction forces between the adsorbate and the adsorbent. According to the nature of the interaction force, adsorption is classified into *physisorption* (physical adsorption) where the binding force is the weak van der Waals force, and *chemisorption* when strong chemical bond between adsorbate and adsorbent forms. Here we restrict our attention to physisorption in this manuscript. Adsorption of methane is a key issues in shale gas development.

One of the main objectives in the study of adsorption is the prediction of the amount of substance that is adsorbed at an interface. Under the equilibrium condition, adsorption is usually characterized by the *adsorption isotherm* which is essentially the adsorbed amount as a function of the ambient pressure at the given temperature^4^. Due to the tremendous complexity associated with both the adsorbate and adsorbent, isotherms can vary greatly under different material and environmental conditions and the determination and analysis of them are mostly done experimentally with aid from theoretical models. Brunauer, Deming, Deming and Teller (BDDT)^5^ qualitatively summarized the experimental physisorption isotherms into five types which form the basis of the modern IUPAC classification^6^ shown in Fig. 1. Type I isotherm is typical of *microporous* (pore widths <2 nm per IUPAC definition^7^) adsorbent while isotherms of Types II and III are typical of *macroporous* (>50 nm) adsorbents with strong and weak adsorbate-adsorbent interactions respectively. Types IV and V occur for strong and weak interactions when the material is *mesoporous* (between 2 and 50 nm) and with capillary condensation. Type VI, added by IUPAC, has steps and occurs for some materials with strong interactions when the temperature is near the melting point for the adsorbed gas^8^,^9^.

Many empirical and semi-empirical adsorption theories have been developed, each based on its own assumptions and can interpret its own subset of the isotherms^10^. Among them, the most widely used ones are the Langmuir equation^11^ derived from kinetic studies based on a mono-layer adsorption model, and the Brunauer, Emmett and Teller (BET) equation^12^ which extends Langmuir’s theory to multi-layer adsorption. The Langmuir theory can interpret the Type I isotherm while Types II and III can be accounted for by the original BET theory and Types IV and V by its generalized version^5^.

Polanyi^13^ assumed that the adsorption of gases on solids is due to an attraction that derives from a potential which is uniquely determined by the spatial position of the gas molecule and therefore independent of the presence of any other molecules in the field of the adsorption potential. In another word, The molecular acceleration process has nothing to do with the environment except the spatial position. It is also assumed that the gas behaves in accordance with its normal equation of state in adsorbed phase. Dubinin assumed that the adsorption force field is independent from temperature^14^.

## Results

**Figure 2:**
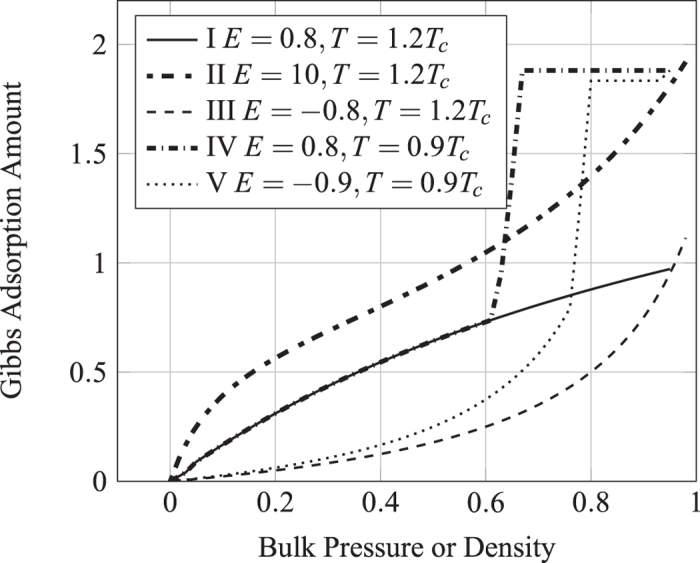
The five adsorption isotherms reproduced by surface-force Shan-Chen model.

A lattice Boltzmann model for physisorption is presented. The adsorbate-adsorbent interaction is modeled via a pseudo-potential in a similar fashion to the non-ideal gas LBM model of Shan and Chen^15^. Various types of adsorption isotherms, both within and outside the IUPAC classification, are observed as the potential is adjusted. Quantitative correspondence with the classic Ono-Kondo theory of adsorption can also be established. When used as a boundary condition, this model compliments the existing LBM methodology and allows adsorption to be simulated together with the hydrodynamic flows of the adsorbate. The model directly associates the LB surface interaction with adsorption energy, and associate adsorption isotherms with Shan-Chen model, which can generate diffusion interface easily. The Shan-Chen model is a method which is very easy to achieve on programming, while introducing Shan-Chen model to adsorption simulation greatly reduces the difficulty of parameters selection for isotherms, as shown in Fig. 2. With this LB model, one just needs to know gas equation of state and the adsorption energy parameter E that can get the right type isotherms within the whole range.

As in the present work, we only considered the adsorption of a adsorbate in a first lattice space. In reality, the situation is almost always affected by the fine structures of the adsorbent. Another important aspect that is not fully treated is the effect of the non-ideal gas. Here we only briefly showed that the adsorbate-adsorbate interaction can add greatly to the complicity of the macroscopic isotherms. It is worth pointing out that both of these additional physics can be handled within the LBM framework and we shall defer the treatments to future works.

## Methods and Discussion

Based on thermodynamics and statistical physics, Ono and Kondo proposed a lattice theory to describe the surface tension and physical adsorption in pure liquids and solutions from the molecular standpoint^16^,^17^. Following Aranovich and Donahue^18^,^19^,^20^, gas molecules are envisioned to reside on a semi-infinite row of sites labeled starting from a solid wall by *i* = 1, ···, ∞. Let *x*_*i*_ be the *occupation fraction* at site *i* which, at the macroscopic level, is the density measured with respect to a maximum density *ρ*_max_, *e.g.*, *x*_*i*_ ≡ *ρ*_*i*_/*ρ*_max_. The molecules interact with those on the nearest-neighboring sites by the interaction energy, *ε*, and with the solid wall by the interaction energy, *ε*_*s*_. The Ono-Kondo difference equation relates the occupation fraction at different layers by:


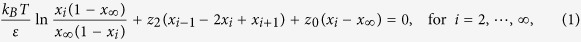


which at *i* = 1 takes the following special form:


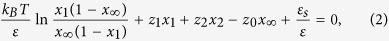


where *k*_*B*_ is the Boltzmann constant, *T* the temperature, *z*_0_ and *z*_1_ the volume and mono-layer *coordination numbers* respectively, and *z*_2_ ≡ (*z*_0_ − *z*_1_)/2. It is apparent that for a given bulk density, *x*_∞_, the density profile is solely determined by the interplay of the interaction energies of *ε* and *ε*_*s*_, both with respect to the thermal energy of the gas particles. Analytical and numerical studies^20^,^21^ of Eqs (1) and (2) indicate that the Ono-Kondo model is able to predict all known types of adsorption behavior^9^. Particularly, at the low-density limit of *x*_*i*_ → 0, Eq. (2) yields:


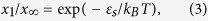


which is Henry’s law as when the density profile drops rapidly to *x*_∞_ away from the wall, the adsorbed amount is solely given by *x*_1_. Next, without the low-density assumption, if the adsorbate-adsorbate interaction can be ignored, *i.e.*, *ε* → 0, Eq. (2) gives:


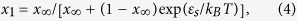


which is the Langmuir isotherm^22^.

While owning its origin to the Lattice Gas Cellular Automaton (LGA) model for fluid mechanics simulation, the Lattice Boltzmann Equation (LBE) can be formulated as a special velocity-space discretization of the continuum Boltzmann equation (c.f., e.g. refs 23, 24, 25, 26). Since the early days of LGA, several attempts were made to include inter-particle interactions in the microscopic dynamics in order to simulate the complex behavior of multiphase flows. A number of schemes were afterwards suggested to incorporate the inter-particle interaction in the framework of LBE at the kinetic level among which, the pseudo-potential theory^15^,^27^ models the van der Waals interaction by introducing a momentum transfer among the particles within an interaction range (nearest-neighbors in most cases) in response to a *pseudo-potential* defined as a function of the local density. The pseudo-potential encapsulates at the microscopic level the details of the interaction that are normally characterized by the pairwise potential in continuum, and at the macroscopic level can be tailored to model many of the effects of a non-ideal gas fluid. Although the pseudo-potential model was first introduced rather intuitively, later analyses linked it to the mean-field theory in continuum^28^,^29^,^30^ by carrying out the BBGKY calculation to the next order.

In this article, we argue that the rich phenomenon of adsorption can be uniformly captured in the pseudo-potential LBE model. By varying the form of the pseudo-potential, a variety of adsorption isotherms manifest in macroscopic numerical simulations. With a few carefully chosen parameters, the wide spectrum of experimentally observed types of isotherms can be numerically reproduced.

The LBE is a special velocity-space discretization of the following Boltzmann-BGK equation:


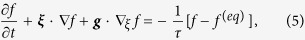


where *f* = *f*(***x***, ***ξ***, *t*) is the single-particle distribution function; ***x*** and ***ξ*** the coordinates in the configuration and velocity spaces respectively; ***g*** the acceleration of externally applied body force, including intermolecular interaction; ∇_*ξ*_ the gradient operator in velocity space; and *τ* the relaxation time. On the right-hand-side is the BGK collision model^31^ with *f*^ (*eq*)^ being the dimensionless Maxwell-Boltzmann distribution^26^:


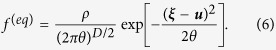


Here, *D* is the spatial dimension; *ρ*, ***u***, and *θ* are the thermo-hydrodynamic variables of density, velocity and temperature respectively, all dimensionless. It has been very well established that Eq. (5) describes the dynamics of a gas consisting of non-interacting particles so that at the various approximation levels, *ρ*, ***u***, and *θ* satisfy the hydrodynamic equations such as the Euler and the Navier-Stokes equations^32^. In statistical mechanics, the Boltzmann equation is formally obtained as the lowest equation of the BBGKY hierarchy with inter-particle correlations completely ignored^33^. The kinetic effects of inter-particle interaction has to be modeled. In the pseudo-potential approach^15^, an interaction force between lattice sites ***x*** and ***x***′ is defined as:


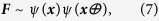


where *ψ*(***x***) ≡ *ψ*(*ρ*(***x***)) plays the role of a *potential* which at the microscopic level dictates the details of the interaction, and at the macroscopic level result in a non-ideal-gas equation of state (EOS)^15^,^27^:


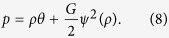


By choosing a proper *ψ*, a wide variety of EOS’s, *e.g.*, those of van der Waals, Carnahan-Starling, and Peng-Robinson, can be simulated^34^,^35^. The interaction force enters into the LBM dynamics by the following momentum increment at each time step and each site:


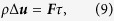


and ***g*** ≡ ***F***/*ρ* corresponds to the external acceleration in Eq. (5).

Interaction between the gas/liquid (adsorbate) and the solid (adsorbent) phases has been introduced into LBM by various authors^36^,^37^. Assuming the form of interaction by Eq. (7) still applies between a “fluid” site, ***x***, and a boundary site, ***x***′. The interaction force exerted on a fluid site in the vicinity of a boundary is proportional to *ψ*(*ρ*(***x***)). To account for the higher complicity in the gas-solid interaction, we allow the pseudo-potential to take a form different from that for the fluid sites, which we shall note by *ψ*_*s*_.

**Figure 3:**
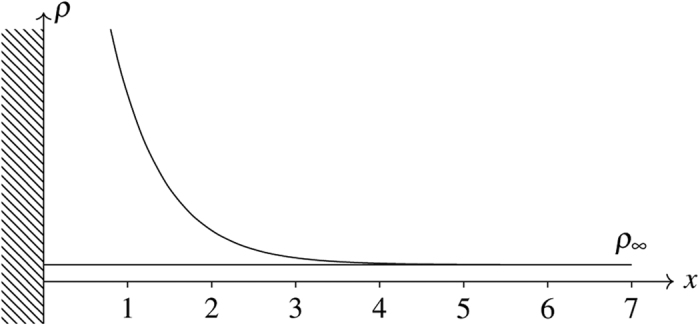
Schematic plot of the one-dimensional lattice. The area under the density profile is the absolute adsorption amount whereas that between the density profile and the *ρ* = *ρ*_∞_ line is the Gibbs amount.

We now investigate numerically the effects of such an interaction on the adsorption isotherms. We note that the amount of adsorption is customarily measured either by the absolute amount or the Gibbs excess amount^38^ which are defined respectively as:


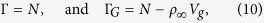


where *N* is the total molar amount of gas, *V*_*g*_ the volume of the gas phase, and *ρ*_∞_ the number density of the bulk gas phase. Our simulation is carried out on a one-dimensional lattice as shown schematically in Fig. 3. The leftmost site, indexed 0, represents the solid wall where the interaction boundary condition is applied. All other sites *i*, 1 ≤ *i* ≤ *K*, are fluid sites with *K* being the index of the last site where we maintain the boundary condition:


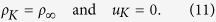


The Gibbs adsorbed amount can be calculated as:


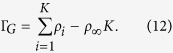


**Figure 4:**
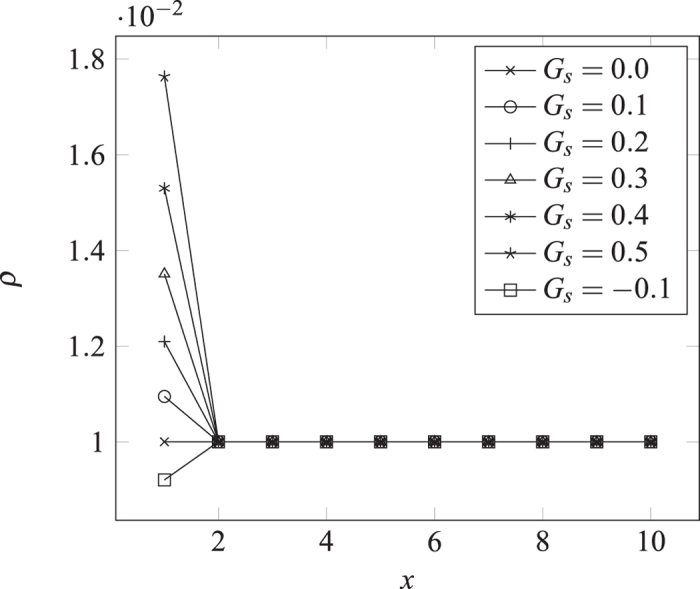
Typical density profile with wall-fluid interaction only and the bulk density set at *ρ*_∞_ = 0.01. To be seen is that the effect of the fluid-wall interaction is confined to the very first lattice site.

Consider first the adsorption in the low-pressure (idea gas) limit in which the adsorbate-adsorbate interaction is absent. For wall-fluid interaction, the simplest model is to have an interaction force linearly proportional to the local density applied only to the first lattice site (nearest-neighbor interaction), *i.e.*:


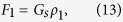


where *G*_*s*_ is a constant interaction strength. Shown in Fig. 4 are the density profiles for a varying *G*_*s*_ with *ρ*_∞_ set at 0.01. To be seen is that only the density at the first lattice is affected. It can be further deduced that the shape of the profile is independent of *ρ*_∞_. By Eqs (9) and (13), Δ*u* = *G*_*s*_*τ*, and consequently the variation in the distribution function due to interaction, is independent of *ρ*_1_. The whole density profile therefore scales linearly with respect to *ρ*_∞_. In particular, the normalized quantity *ρ*_1_/*ρ*_∞_ is a function of *G*_*s*_ only but independent of *ρ*_∞_. We can therefore write:


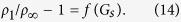


**Figure 5:**
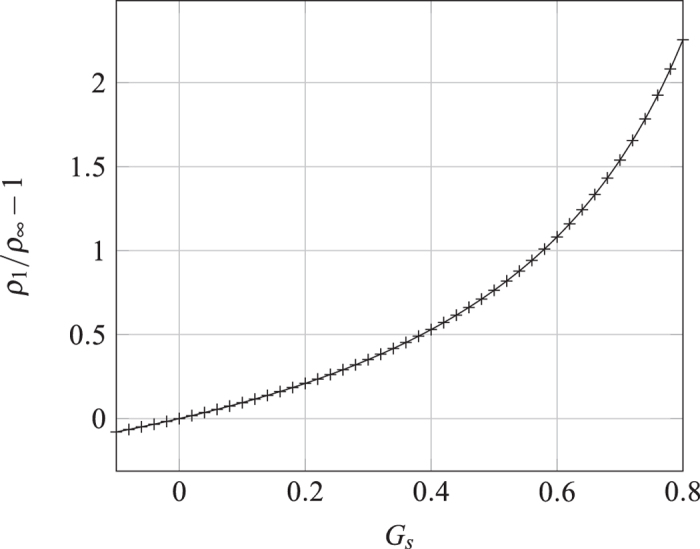
The normalized Gibbs amount *vs.* the interaction strength obtained by numerical simulation. Note that the function form is independent of *ρ*_∞_.

Shown in Fig. 5 is the function *f*(*G*_*s*_) obtained in numerical simulation with its independence on *ρ*_∞_ verified.

A number of observations can be made. First, the left-hand-side of Eq. (14) is essentially the *normalized* Gibbs amount which should vanish in the absence of wall-fluid interaction. The numerical model correctly reproduces this feature. Second, *ρ*_1_ is linear in *ρ*_∞_, which is Henry’s law. Third, comparing Eq. (14) with Eq. (3), the following relation between the present LBM model and the Ono-Kondo lattice model can be identified:


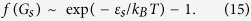


Beyond the low-pressure limit, the more complex interaction is modeled *via* a pseudo-potential as in the bulk of the fluid^15^. To facilitate comparison with the Ono-Kondo theory, we rewrite Eq. (4) as:


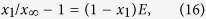


where:


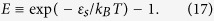


Apparently Henry’s law (Equation (3)) is recovered as *x*_1_ → 0. Noticing *f*(*G*_*s*_) ≅ *G*_*s*_ at small *G*_*s*_, and comparing the equation above with Eq. (14), we let *G*_*s*_ ~ 1 − *ρ*_1_ and extend the linear force model of Eq. (13) by:


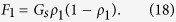


**Figure 6:**
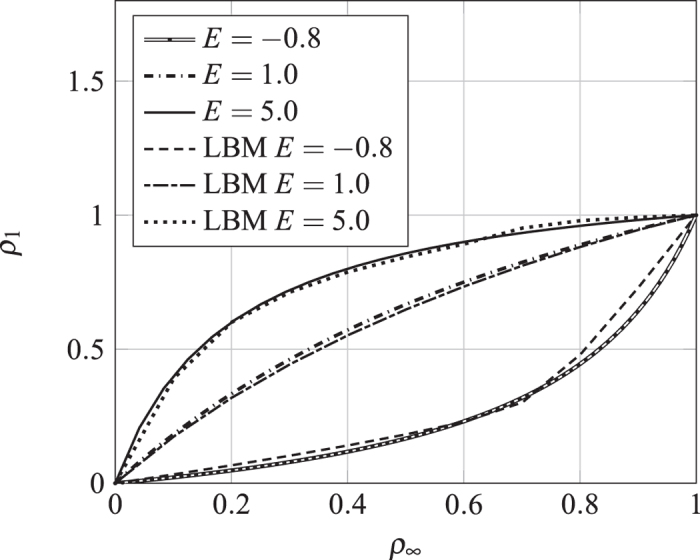
Comparison between the isotherms obtained in the LBM simulations with those predicted by the Ono-Kondo theory. The lines are the solutions of Eq. (4) for different values of *E*.

Shown in Fig. 6 are the isotherms obtained from LBM simulation and Eq. (16). To be seen is that the LBM results agree well with the Ono-Kondo theory if the interaction is weak. The deviation at stronger interaction can be attribute to the approximation *f*(*G*_*s*_) ≅ *G*_*s*_. The discrepancy for *E* = −0.8 for high values of *ρ*_∞_ can be eliminated with simple approximate correction function. Depend on the signs of *E* or *G*_*s*_, the isotherms exhibits the features of Types I and III respectively.

**Figure 7:**
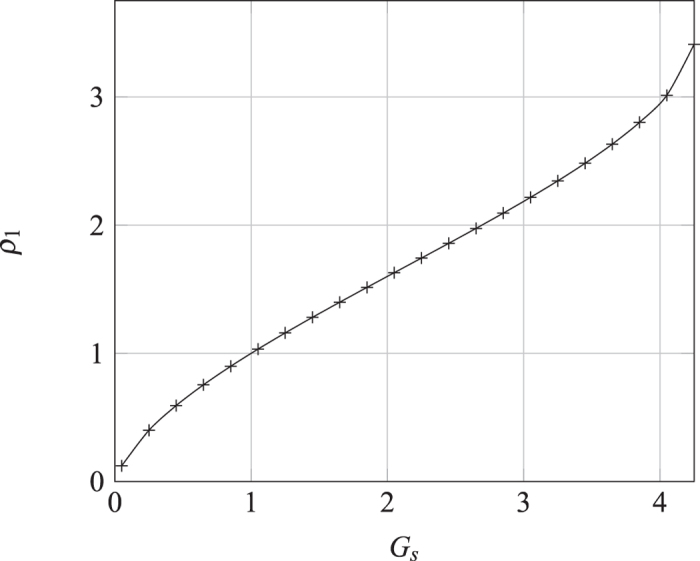
Adsorption isotherm using the force model of Eq. (19). Certain features of Type II isotherms are exhibited.

Furthermore, isotherms with more interesting features can exhibit in the macroscopic level if more complicated pseudo-potential functions are used. Shown in Fig. 7 is a typical isotherm for the interaction:


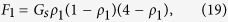


which has the typical Type II features. Adsorbate-adsorbate interaction can be achieved by means of gas equation of state (EOS). The experimental data of diverse fluids analyses show near their critical point all fluids exhibit similar properties, so ratios of pressure, temperature and density of the absolute value and critical value can be used to export real gas equation of state. These ratios allow all parameters be dimensionless.


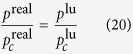



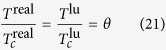



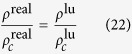


**Figure 8:**
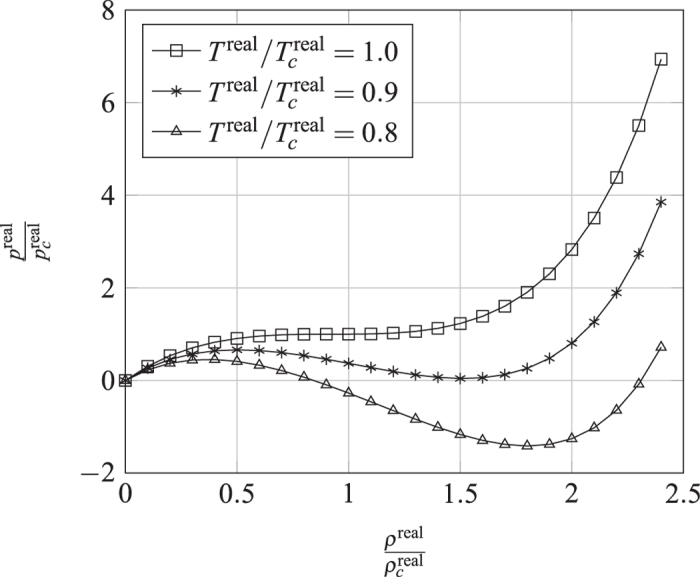
P-R EOS system under the lattice units in Eq. (25). 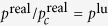
, 
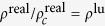
.

**Figure 9:**
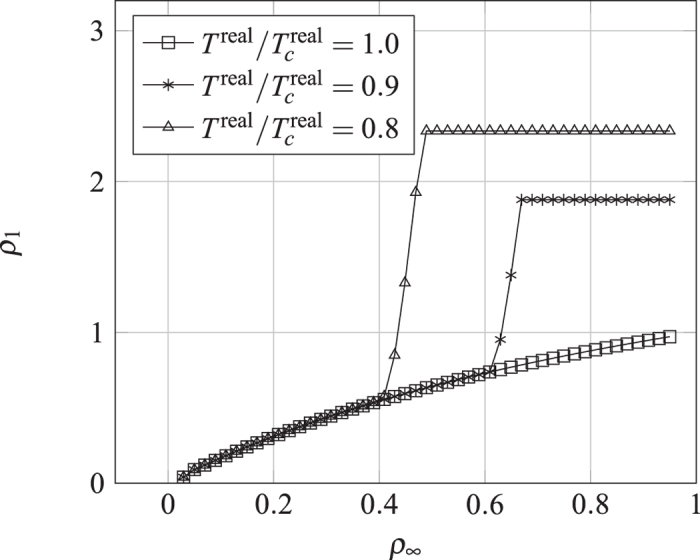
The adsorption isotherm generated by P-R EOS in Fig. 8, *E* = 0.8.

For convenience, set lattice critical pressure 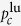, critical temperature 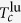 and critical density 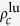 to 1, thus, equation of state is presented by the potential function:


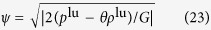



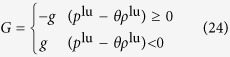


where *g* is a positive figure denoting the strength of the interaction force. Peng-Robinson^39^ (P-R) EOS is shown in Fig. 8:


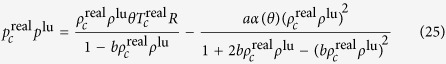



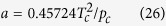



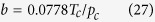



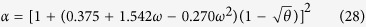


where *ω* is the acentric factor of the species, for methane is 0.008. Insert Eqs (25, 26, 27, 28) in Eq. (23) to obtain *ψ*’s expression. Under the P-R EOS, the Eq. (18) still holds, and then it can simulate a non-ideal gas adsorption isotherm, as shown in Fig. 9.

**Figure 10:**
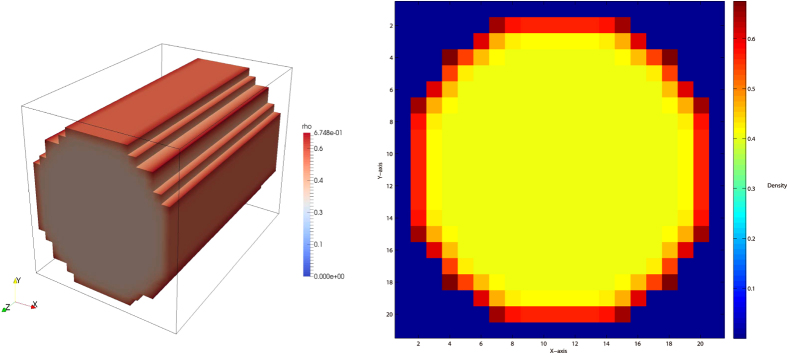
The adsorption density distribution on three-dimensional D3Q19 lattices tube with a size of 21 × 21 × 30. 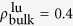
, *E* = 1.0, 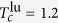.

On the surface, if the temperature below the critical temperature, the condensation occurs, resulting in liquid film, *E* > 0 yield type IV isotherm, *E* < 0 yield type V isotherm, as shown in Fig. 9. When condensation does not occur, isotherm is mainly controled by *G*_*s*_, but once condensation occurs, liquid film will soon occupy the entire surface, and the growth rate of adsorption amount in small pores reduce. Figure 9 can be seen as an adsorption isotherm in very close parallel plate, which can also be regarded as the micro pores. If the temperature is over the critical temperature, *E* > 0 yield type I isotherm, and *E* < 0 yield type III isotherm, and Eq. (19) yield type II isotherm. Since this model based on pseudo-potential, it is easy to be extended to three-dimensional. We use D3Q19 to achieve three-dimensional adsorption simulation, as shown in Fig. 10. Here a standard bounce back no-slip boundary condition is used to ensure conservation of mass. The adsorption is currently addressed in LBM as mass transfer process without directly linking to isotherms^40^,^41^, while our model can be used to reproduce five types of isotherms and gives the relationship between them and the adsorption energy. In addition, we also noted the new progresses on the EOS and interaction of fluid-solid interfaces^42^,^43^,^44^,^45^.

## Additional Information

**How to cite this article**: Guo, L. *et al*. Modeling adsorption with lattice Boltzmann equation. *Sci. Rep.* **6**, 27134; doi: 10.1038/srep27134 (2016).

## Figures and Tables

**Figure 1:**
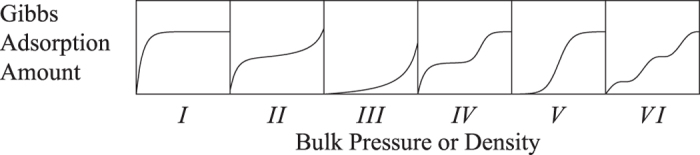
The IUPAC recommended sub-critical adsorption isotherms classification.
